# Progress in Otology

**Published:** 1895-11-30

**Authors:** 


### Progress in Otology.

(*Continued from page* 136.)

**The Dry Treatment of Otitis Media Suppurativa.**—Nichols[16] writes upon a plan of "dry" treatment, which he considers superior to what is generally understood by that designation (Bezold's). Although certain cases not of long standing or serious character may have been cured by the older method, the usual result, he says, is the formation of a solid plug of dried boric acid pus and epithelium, which dams up the canal until forced out by the discharge or removed by the patient; in addition to which there is a desquamating effect on the skin of the meatus. Nichols supplies his patient with an "ear-kit," which consists of a box containing a soft rubber "Ideal" ear syringe, a bottle of hydrogen peroxide, a package of absorbent cotton, a dropper, and some wooden toothpicks. The directions are to first syringe out the ear thoroughly with plain warm water, then to turn the head with the affected ear uppermost, and fill the canal from the dropper with the peroxide. This is allowed to remain until bubbling ceases. The ear is then syringed out again, and the peroxide used as before. This process is repeated several times, until the peroxide fails to act. A small mop is then made with the cotton and a toothpick, and the canal mopped repeatedly until the cotton comes out dry. The mops must be small, and the end of the pick well protected, so as to avoid injuring the parts. This cleansing process requires at least fifteen minutes for its performance, and must be repeated two or three times a day, according to the activity of the discharge. If profuse, the patient is directed to carry cotton in his pocket, and at frequent intervals during the day mop the canal dry. A few other points in this paper may be noted. When the perforation is small it must be enlarged with a galvano cautery point or a sharp-pointed bistoury. Cocainisation, preparatory to this, is best performed by placing against the membrane a small ball of solid benzoinol with 20 per cent. cocaine. Granulations must be removed. For large ones the best applications are iodine and carbolic acid equal parts, nitrate of silver 50 per cent. solution, or liq. ferri perchlor. Orthochlor-phenol is also very useful. Carious bone should be curetted away, and touched with aromatic sulphuric acid. Of alcoholic solutions alumnol, five grains to the ounce, is preferred to boric acid in alcohol. Iodoform and its substitutes set up dermatitis, and are not to be recommended.

**Septicæmia following Middle Ear Disease.** — Gifford Nash[17] reports (1) a case of a boy aged twelve who had had otorrhœa for five years, and was boxed on the ear on June 16th, 1892. Immediately after this, there was headache, followed later by vomiting, and twenty-four hours from the date of blow he was lying curled up in bed, objecting to light and interference, with a temperature of 103, and pulse 180. Under chloroform, a small granulation polypus was syringed out of the right ear, and leeches and the ice-bag were applied. On the 18th there was fever, but no delirium. The mastoid antrum was now exposed by operation and the dura mater covering the roof was examined and found healthy. On the 20th, symptoms of pneumonia set in, and by the 22nd the temperature was 104. Friction was heard in conjunction with tubular breathing, and there were all the symptoms of septic pleuro-pneumonia. Having decided to explore the lateral sinus, the mastoid was trephined over it, and the sinus exposed. A hypodermic syringeful of fluid blood was withdrawn from the sinus, so the wound was closed and nothing further was done. From June 22nd till the end of July, the case was desperate; both lungs became involved, the expectoration was fœtid, and the emaciation extreme. The patient gradually recovered, and was out of doors by October 1st. Treatment consisted of milk, eggs, and brandy, with quinine and digitalis. He became quite well, discharge from the right ear ceased, and the watch-tick was heard at twelve inches. Nash thinks it would have been better to have ligatured the internal jugular and cut short the illness by screening the lungs. The sequence of events he believes to be as follows: Pleuro-pneumonia from blow on the head, dislodging septic thrombus from middle ear, and producing thrombosis of some vein entering the lateral sinus. Source of the sepsis—sewer gas—the house being in a very insanitary state. (2) Case of extra-dural abscess and thrombosis of lateral sinus: The patient was a boy aged fifteen, who had left-sided otorrhœa for two years, a polypus having been removed from a perforation in the membrane the previous summer. He was first seen on April 1st, 1894, when the symptoms of sinus disease were well marked. The middle fossa of the skull was opened with a trephine, and the dura mater was found healthy, but on exposing the antrum, the bone behind was found to be very soft, and on gouging it away, an abscess, lying between the bone and the lateral sinus, containing 2 drs. of foul pus, was evacuated. A free escape for this was provided, and next day the internal jugular was tied, the wound re-opened, the lateral sinus (the walls of which were sloughing) cut away, and a firm dark blood clot turned out. Later on it became necessary to again re-open and enlarge the mastoid wound, remove granulations, and let out about 2 drs. of pus from the occipital portion of the lateral sinus. A drainage tube was inserted in the sinus, and the middle ear thoroughly scraped out. After one more complication, consisting of an abscess in the neck which had to be opened, all went well with the patient, and by May 16th the wounds were healed. The sequence here was (1) chronic otorrhœa and suppuration in the antrum, extra-dural abscess, cleared out and drained; (2) septic involvement of lateral sinus, ligature of internal jugular to cut off absorption, opening of lateral sinus to permit escape of breaking down clot; (3) subsequent operation to let out pus from sinus; and (4) opening subcutaneous abscess in the neck.

[16] Amer. Med.-Surg. Bull., May, 1895. [17] Lancet, Aug. 3, 1895.

